# Facing distortion: Impact of spatial distortions on upright and inverted face identification

**DOI:** 10.1371/journal.pone.0308675

**Published:** 2025-09-23

**Authors:** Bliss Cui, Peter Bex

**Affiliations:** Department of Psychology, Northeastern University, Boston, Massachusetts, United States of America; University of Canberra, AUSTRALIA

## Abstract

Face identification is a critical activity of daily living that may be impaired by blur or distortions caused by vision loss or prosopometamorphopsia. In this study, we examine the face inversion effect as a benchmark for understanding how distortions impact the identification of upright and inverted faces. Bandpass-filtered noise (F_peak_@1–32 cycles/face) was used to generate pixel shifts to distort calm and neutral faces from a standardized face database. The amplitude of distortion was varied using an adaptive staircase. 8 normally sighted subjects were given unlimited time to identify which of 4 distorted faces matched the identity of an undistorted reference face, each presented in a 6.5°-9.8° ellipse in a 2*2 grid. Image cues were removed from each face by equalizing them to the luminance distribution and chrominance of the average face. There was a significant interaction between face orientation and distortion frequency (F5,35 = 2.72, p = 0.0354), where sensitivity as a function of distortion frequency monotonically increased for inverted faces but was asymetrically U-shaped with a vertex at 4–8 cycles/face for upright faces. For upright faces, thresholds were lowest at mid spatial frequencies, with significant differences (p < 0.05) observed at the highest frequencies (16 and 32 cpi), supporting an asymmetric U-shaped tuning profile. In contrast, thresholds for inverted faces increased progressively across spatial frequencies, consistent with a monotonic trend. These results suggest that upright face recognition is most impacted by distortions at mid frequencies, whereas inverted face recognition declines more linearly as spatial frequency increases. The peak distortion frequency is correlated with the distance between the eyes, consistent with a critical role for eye geometry in upright face identification. These results suggest that the face inversion effect for distortion is selective for high-frequency bands.

## Introduction

Face identification is a critical activity of daily living in workplace interactions and social settings, and deficits in face processing are associated with a broad range of psychosocial problems [1]. Face processing is specialized and utilizes a large, dedicated set of neural regions including the fusiform face area for identity [2,3] and the amygdala for emotion [4]. Difficulty in face processing is seen in disorders like prosopagnosia and prosopometamorphopsia [5,6].

Prosopagnosia is the inability to recognize familiar faces, or “face-blindness” [7,8] and is a selective visual agnosia that can be acquired or developmental [9]. The striking feature of this disorder is its selectivity towards faces and not other visual things like objects or words [7]. This property is widely recognized as evidence of our human brain’s specialized face-processing abilities [6].

Prosopometamorphopsia occurs in 1.5−1% of the population, and is the perception of distortions only when viewing faces and not other objects [10]. Faces may appear to warp, and facial features may scramble [10], causing major hindrances in facial recognition. It has comorbidities with disorders like age-related macular degeneration [11], a highly prevalent disease that affects 196 million people worldwide and is projected to increase to 288 million in 2040 [12]. Face identity recognition within prosopometamorphopsia has not been thoroughly investigated. However, a recent study tested familiar (well-known celebrities) face recognition using distortion and a parametric model of prosopometamorphopsia [13]. They concluded that face recognition performance varies systematically with the parameters of distortion employed and that the effects scale with face dimension rather than viewing angle [13]. While their study involved familiar faces, it did not directly compare performance with unfamiliar or inverted faces. Our study focuses on unfamiliar faces to isolate perceptual effects of the spatial scale of distortions without the influence of prior knowledge or memory. Unlike their emphasis on naturalistic variability, we employ controlled warping techniques to directly assess how distortions at distinct spatial frequency bands impact recognition. These complementary approaches provide insight into different aspects of face processing, with our study emphasizing the perceptual mechanisms underlying distortion sensitivity and theirs highlighting the role of familiarity in modulating these effects.

Distortions alter the spatial relationships among image features but are difficult to quantify in clinical populations [14]. The standard method to detect the presence of visual distortion involves subjective reports with Amsler grids [15] which are unreliable [7,14,15]. Amsler grids are diagnostic tools used in ophthalmology to detect and monitor visual distortions, often associated with macular degeneration [7]. Several methods have been developed to quantify distortion, including M-CHARTS [16], preferential hyperacuity perimeters [17], and shape discrimination tasks [14,18]. However, pathological distortions are highly idiosyncratic [14], so several groups have developed methods to simulate visual distortions as an effective way to alter image structure in a uniform and controlled manner while leaving the luminance, chrominance, and spectral properties relatively unchanged [13,19,20], unlike other methods such as the addition of noise or manipulations of size, contrast or blur. These methods employ image-warping techniques to shift and fill in image regions and allow control over the spatial scale (how often spatial distortions repeat per unit of distance) and the magnitude of distortion.

**Fig 1:**
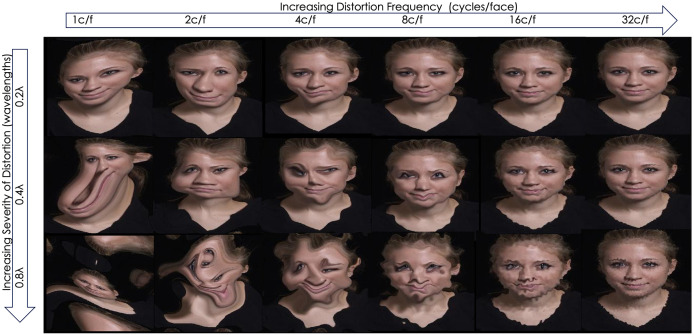
Scale of spatial distortion. The distortion frequency (x-axis) represents the frequency of cyclic variation. The severity of distortion (y-axis) is the magnitude of displacement. This creates changes in the configural and featural information in a face.

Such distortions modify the relative positions and shapes of face features at different scales, as illustrated in Fig 1. Low spatial frequency distortions, (left column of Fig 1) predominantly change the configuration of face elements, such as the position of the nose relative to the eyes, with limited effect on their shape. High spatial frequency distortions (right column of Fig 1) primarily modify the shapes of smaller features (such as the nose) with limited effect on their configuration within the overall face. Both featural and configural information contribute to the processing of human faces [21]. Featural information refers to specific local features on the face like the eyes, nose, and mouth. Configural information is extracted from the geometric relationship between two or more features together, for example, the distance between the eyes. Thus, depending on the spatial scale, visual distortions alter primarily the configural, the featural elements of faces, or both. A study investigated which spatial frequencies supported configural and featural processing [22]. To manipulate the configural properties of faces, they adjusted interocular distance and eye heights, and to manipulate the featural properties of faces, they replaced the original eyes with eyes from another face. The main takeaway was that low spatial frequencies mainly support configural processing and high spatial frequencies support featural processing.

This featural-configural distinction has been influential in guiding studies of face recognition and is particularly relevant in contexts where visual information is degraded or disrupted. However, it is increasingly recognized that this binary framework is a simplification, as both types of information are often processed in parallel and interactively [23,24]. Moreover, the way specific types of image distortions affect these perceptual processes may not map cleanly onto this dichotomy. In the present study, we use this framework as a starting point to explore how spatial frequency-based distortions influence face recognition, while remaining cautious about its limitations and open to more nuanced interpretations.

One of the most investigated phenomena in the face perception literature is the Face Inversion Effect (FIE) [25], where the identification of faces is disproportionately hindered when faces are inverted as opposed to upright [25,26]. The FIE is thought to be caused either by innate face-specific mechanisms [2,27], although the evidence for FIE effects in non-human primates is weak [28]; or by a consequence of expertise with faces [26,29], and is probably due to both factors [30]. The FIE is thought to arise from two complementary factors: (1) the development of face-specific perceptual mechanisms through early and extensive experience with upright faces during infancy and childhood (ontogeny), and (2) the evolution of specialized neural systems for face processing in humans (phylogeny). Developmentally, research indicates that infants as young as a few months old begin to show a preference for upright faces, and this sensitivity strengthens over the first year of life, suggesting that experience plays a crucial role in shaping face processing abilities [31–33]. Evolutionarily, comparative studies have shown that while some non-human primates, such as chimpanzees, exhibit a FIE for conspecific faces, the effect is generally weaker and more variable across species, indicating that the robust FIE observed in humans may be a result of evolutionary adaptations specific to human social cognition [28]. Therefore, the FIE likely reflects both the ontogenetic development of face expertise and phylogenetic specialization of face processing systems in humans [34]. Both hypotheses posit that the FIE is evidence for configural processing since face inversion impairs configural information more than featural information. Several studies have shown that configural changes are easier to detect when faces are upright rather than inverted [35]. One of the clearest demonstrations of the FIE is the famous Thatcher illusion, where the inversion of the eyes and mouth are undetectable when the face is inverted, but glaringly obvious when the face is shown upright [18]. In one study, Boutsen and colleagues [36] used electroencephalogram (EEG) to observe event-related potential (ERP) correlation on objects, faces, and “Thatcherized” faces. They found that all their results were face-specific, and that face and feature inversion are distinguished on both a functional and neural level [36]. We use the FIE as a benchmark to assess how spatial distortions affect face recognition under typical (upright) and altered (inverted) viewing conditions.

This study has 2 goals: first, we aim to determine how the spatial scale and amplitude of distortions affect the identification of controlled, unfamiliar faces. Second, we compare the tuning of distortion for upright and inverted faces to examine the spatial dependence of the FIE.

## Materials and methods

### Participants

There were 8 naive subjects who participated in the experiment, including 3 females and 5 males with a mean age of 25.5 years. Power analyses from similar paradigms in the face processing literature [13] suggest that our sample size and trial count are appropriate for detecting medium to large effects. All participants had normal or corrected-to-normal vision. The recruitment period was 11/1/2021 to 11/19/2021. This study was not pre-registered. The Northeastern Institutional Review Board approved this study that obtained written consent from human participants: Psychophysical Study of Visual Perception and Eye Movement Control (IRB 14-09-16).

The face images used in this study were obtained from the Interdisciplinary Affective Science Laboratory (IASLab) Face Set [37], a publicly available database, designed for academic research. Permission to use these images for this study was granted. The IASLab Face Set was developed with support from the National Institutes of Health Director’s Pioneer Award (DP1OD003312) to Lisa Feldman Barrett.

### Stimuli

Stimuli were displayed on a 32-inch LG UHD (3840 x 2160) 60 Hz display HP at a viewing distance of 60 cm and subtended 60.5° * 34°. The experiment was programmed and run in Matlab 2021a within Psychtoolbox-3 [38].

Faces were selected from the IASlab Database [37] of 51 unique identities (32 female and 19 male). This database includes standardized poses of each identity displaying neutral, sad, happy, angry, fearful, surprised, calm, excited, and disgusted facial expressions. In addition, all poses have a direct gaze and averted gaze variants and open mouth and closed mouth variants. All images have been edited so that the eyes are centered in the same location. The database appears to contain some racial/ethnic diversity. A subset of expressions (forward gaze, closed mouth examples) of the IASLab Face Database has been normed by ratings of emotion category, attractiveness, and intensity. For our purposes, we used calm and neutral expressions with forward gaze and closed-mouth poses. We chose not to remove any faces with easily identifiable features because we did not want to introduce any bias. To preserve ecological validity, we retained external facial identity cues such as hairstyles and facial contours. These cues are known to contribute substantially to face recognition in everyday contexts [39–41] Excluding them may have imposed artificial constraints on participants’ processing strategies, leading to a less naturalistic mode of face perception and potentially biasing the interpretation of spatial frequency effects.

### Image processing

We applied a visual distortion to faces based on a method previously used for both letter recognition [14] and as a model of the symptoms of prosopometamorphopsia [13]. Distortions were created with pixel shifts and image warping. Pixel shifts were controlled with bandpass filtered white noise (raised cosine isotropic filters in the frequency domain with 1-octave full-width half-height bandwidth,


$$\text{0},\;\omega <\omega_{\text{peak}}-\text{1}$$

$$\text{h}(\omega )=\{\text{0}.\text{5}*(\text{1}+\text{cos}(\pi *(\omega -\omega_{\text{peak}})),\;\omega_{\text{peak}}-\text{1}<\omega >\omega_{\text{peak}}+\text{1}$$

$$\text{0},\;\omega >\omega_{\text{peak}}+\text{1}$$

where ω is spatial frequency and ω_peak_, peak spatial frequency, was 1–32 cycles/face in 6 equal log steps. Independent noise samples were used for horizontal and vertical shifts, and new filtered noise samples were generated for each face on each trial. The sign and amplitude of the band-pass filtered noise determined the position shift of each pixel in the face image. Fig 1 displays the scale of spatial distortion used in the experiment. The X-axis shows increasing distortion frequency. The magnitude of distortion was controlled by scaling the noise and the effect of amplitude is illustrated in Fig 1 increases on the y-axis.

Unlike manipulation methods such as blur or contrast, distortion does not alter image chrominance and luminance statistics, although it may alter the spatial frequency by an undetectable amount [19].

The amplitude of distortion was varied using an adaptive staircase in which distortion amplitude was decreased by 1/3 dB after correct trials and increased by 1dB after incorrect trials [42]. The amplitude on the first trial was fixed by the experimenter based on pilot data. This prevents the experiment from being too easy or too difficult and results in a quantifiable numerical distortion threshold that is unique to one’s ability to recognize faces under distortion.

**Fig 2:**
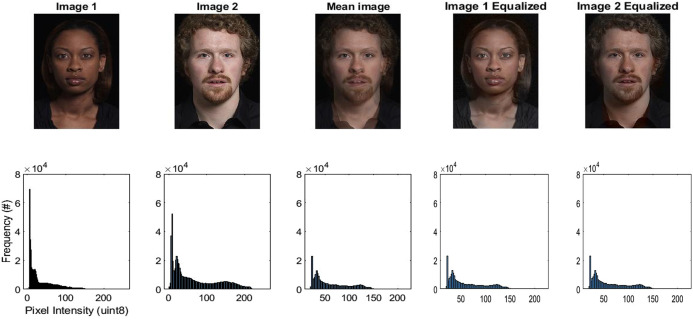
Matching image chrominance and luminance. Images of different people were selected at random from the IASL Database. A histogram of luminance is shown below each image. Pixels from image 1 and 2 were sorted in ascending order. The mean image was created with the combined/averaged luminance and chrominance information, and this was reapplied onto the original image to create an equalized image.

The 4 distorted faces were each presented in a 6.5°-9.8° ellipse, arranged in a 2*2 grid. To generate the average face used in the analysis, we randomly selected 50 identities from the IASLab face set. For each identity, one front-facing image labeled as “neutral” or “calm” was selected. Each image was resized to 600 × 400 pixels if necessary, and pixel values were summed and averaged across all images to create a single composite face. The resulting image was converted back to 8-bit format for visualization and analysis. Image cues were removed from faces by equalizing the luminance distribution and chrominance of the computed average face values in each individual trial. Fig 2 shows an example of how the luminance and chrominance statistics of face images were equalized. The top row shows example face images, the bottom row shows histograms of the luminance values with pixel intensity on the x-axis and frequency on the y-axis. Images 1 and 2 show two different example faces from the IASLab database [37], the Mean image is the pixel-wise mean of the 2 faces. The luminance and chrominance of the source and mean images are then extracted with MATLAB’s function rgb2ycbcr() [43]. The luminance distribution of the source images (y plane) is swapped in ascending order with the luminance distributions of the mean image, and their C_b_ and C_r_ planes are replaced with those of the mean image. The equalized versions of images 1 and 2 are converted to RGB images with ycbcr2rgb() to generate images resembling the source images but with identical luminance and chrominance distributions. This technique prevents the identification of a face based on luminance or chrominance properties alone and also minimizes bias in choosing a face due to skin color and maximizes inclusivity.

### Procedure/Task

**Fig 3:**
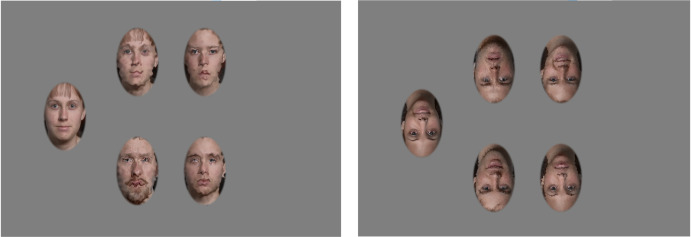
AFC task. Upright and Inverted Face ID Task displayed the undistorted target face on the left and 4 distorted faces centered on the screen including the matching face and 3 distractor faces. Participants were prompted to choose the face that matched the identity of the person displayed on the left.

The participants were seated at a viewing distance of 60 cm. For each trial, they were prompted to choose which of the 4 distorted faces matched the identity of the undistorted target face on the left side of the screen (shown to scale in Fig 3). Subjects commenced the experiment by clicking anywhere on the screen with the mouse. The cursor was centered at the beginning of each trial on a gray background. 1 undistorted target face was displayed on the left. 4 distorted faces were centered on the screen including 3 distractor faces and 1 face with the same identity as the target, but a different image to prevent point wise matching. These photos were randomly selected from the IASlab database of faces from the calm/neutral expressions. The 5 Distortion Spatial Frequencies were randomly interleaved in each run, and the distortion magnitude was controlled by a staircase. Participants were given unlimited time to select their choice. Subjects were not given the option to change their choice to encourage mindfulness before selection. When the observer clicked on their face of choice, a high or low pitch tone was played, indicating a correct or incorrect choice respectively. The cursor was recentered to avoid response bias, and the next trial began. There were 40 trials x 5 distortion spatial frequencies resulting in 200 total trials per observer.

This entire process was repeated to compare the spatial frequency tuning of distortions for inverted faces. The order of upright and inverted tasks was randomized to counterbalance the learning effect bias. Participants were given a break in between tasks. The methods were the same as for the upright tasks except that all faces were rotated 180 degrees.

### Power analysis

This study was modeled after Dear and Harrison (2021), who used a similarly small sample size (n = 7) in a repeated-measures design with high trial counts per condition. Although our sample size was limited (n = 8), the within-subjects design provided strong statistical sensitivity to large effects. A post hoc sensitivity analysis was conducted using G*Power [44] for repeated-measures ANOVA (α = 0.05, power ≈ 0.80), confirming that the design was adequate for detecting large effects. Effect sizes were calculated using partial eta squared (η^2^ₚ). For the main effect of Face Orientation, η^2^ₚ = 0.597 (Cohen’s f ≈ 1.22), and for the Face Orientation × Lambda interaction, η^2^ₚ = 0.39 (Cohen’s f ≈ 0.80), both indicating large effects. While the study may be underpowered for detecting smaller effects, it was appropriately designed for the exploratory identification of robust perceptual differences. We acknowledge the limited generalizability due to sample size and encourage future studies to replicate these findings with larger and more diverse samples.

## Results

**Fig 4:**
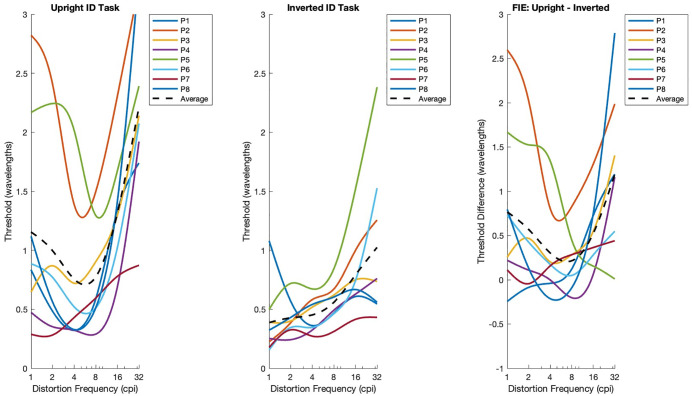
Distortion tolerance graphs. The X-axis displays the Distortion Frequency in Cycles per Image (CPI), and the Y-axis displays the Tolerance to Distortion in wavelengths. Participants are color coded, with the average tuning curve shown in black. 4a) Shows the unique average tuning curve for the Upright Identification Task. 4b) Is the Inverted Identification Task. 4c) Displays the difference between the upright and inverted tasks, representing the Face Inversion Effect (FIE). The FIE is absent at the middle frequencies.

Fig 4 shows threshold distortion magnitude as a function of peak distortion spatial frequency. Threshold distortion amplitude is the distortion magnitude at which observers were able to select the distorted face that matched the undistorted target face in 62.5% trials. Threshold distortion is expressed in multiples of the distortion wavelength. Individual tuning curves were generated by fitting smoothing splines to each participant’s distortion threshold data across spatial frequencies. The x-axis frequencies were log-transformed to provide even spacing, allowing the spline fits to produce smooth, continuous curves that capture the overall tuning trend while reducing noise. In this 4AFC task, the guessing rate was 25%. There was a monotonic relationship between distortion magnitude and error rate, such that errors increased systematically as distortion magnitude increased. Note that a high threshold indicates that observers could identify faces that were subject to extreme distortions (i.e., distortion-resistant), whereas a low threshold indicates that face identification is impaired by mild distortion (i.e., distortion-vulnerable). Data show the threshold distortion amplitude as a function of distortion spatial frequency for upright faces and inverted faces. The red horizontal line shows the mean threshold for 8 observers, error bars show + /-1 standard error, and the lines show the best fitting 2nd order polynomial.

To ensure that the observed tuning effect was not driven by averaging across participants, we included individual tuning curves for each participant. The results show that tuning is pretty consistently present across individuals, confirming that the effect is not limited to a subset of participants but rather reflects a systematic pattern in face recognition under spatial frequency distortions. This strengthens the conclusion that the tuning effect is a robust characteristic of face perception, rather than an artifact of group-level averaging.

For upright faces, thresholds measured in wavelengths were lowest at mid frequencies (4–8 cycles per image), resulting in a distinctive asymmetric U-shaped tuning curve, and indicating that upright face recognition is most sensitive to distortions in this range. A two-way repeated-measures ANOVA with Face Orientation and Distortion Frequency as within-subject factors revealed significant main effects of Face Orientation (F1,7 = 13.18, p = 0.0084) and Distortion Frequency (F5,35 = 20.57, p < 0.0001), as well as a significant interaction between orientation and frequency (F5,35 = 2.72, p = 0.0354). This interaction reflects the U-shaped pattern for upright faces versus the monotonic increase for inverted faces.

To further examine the face inversion effect, paired t-tests were conducted at each distortion frequency. These tests showed significant inversion effects at 16 and 32 cpi (t7 = 3.23, p = 0.0145; t7 = 3.68, p = 0.0079, respectively). The mean threshold difference between upright and inverted faces at mid frequencies (4–8 cpi) was close to zero, indicating minimal FIE in this range. These results suggest that the magnitude of the face inversion effect depends on spatial frequency, being strongest at high frequencies and absent at mid frequencies, consistent with frequency-specific tuning of upright face recognition.

To summarize, higher thresholds reflect greater tolerance to distortion and better performance. For upright faces, identification was most impaired by distortions in the 4–8 cpi range, whereas inverted face recognition showed no clear dependence on spatial frequency, with similar impairment across all distortion levels. This produced an asymmetric U-shaped tuning curve for upright faces, with the largest face inversion-related performance drop occurring at mid spatial frequencies.

## Discussion

This study explored how the spatial scale and amplitude of distortions affect face identification in upright and inverted faces. The results identified significant differences in the impact of distortions on upright and inverted faces, thus replicating the Face Inversion Effect (FIE) in a novel paradigm. There was a significant interaction between face orientation and distortion frequency: participants tolerated greater distortion when identifying upright faces, indicating higher resistance to distortion. This observation is consistent with the FIE: subjects are better at identifying upright faces than inverted faces.

Additionally, the tuning curves for face matching thresholds as a function of distortion spatial frequency differed for upright and inverted faces. Upright face thresholds showed an asymmetric U-shaped profile, with the greatest sensitivity at mid spatial frequencies (4–8 cpi), whereas inverted face thresholds increased more monotonically with frequency. Paired comparisons between upright and inverted faces at each frequency revealed a significant face inversion effect (FIE) at high frequencies (16–32 cpi) but not at mid frequencies (4–8 cpi). This indicates that, although upright face recognition is most disrupted by distortions in the mid-frequency range, the FIE itself is minimal there and emerges primarily at higher spatial frequencies. These results suggest that the FIE for distortions is selective for certain spatial frequency bands.

The spatial frequency (SF) range in which distortions most strongly disrupted face identity recognition in our study (4–8 cycles per image, cpi) is notably lower than the 8–16 cpi range reported in prior studies [25,45]. This discrepancy may be attributed to differences in methodology. Whereas prior research often employed filtered images or added noise at specific frequency bands to isolate SF contributions, our study utilized a warping-based distortion method. This approach may have introduced broader alterations to both global and local properties of the face, including spatial relationships and orientation content, which could shift the effective SF range influencing performance. Additionally, individual differences in SF sensitivity or task-specific demands, such as the reliance on matching distorted stimuli rather than naturalistic faces, may also contribute to the observed difference. Future research could explore whether the observed shift in SF tuning is a result of methodological differences or represents a distinct characteristic of the warping paradigm used in this study.

The critical frequency of 4–8 cpi is correlated with the distance between the eyes, among other features. This was determined by computing the average face, estimating the distance between the centers of the eyes, estimating the size of the face, and calculating the ratio of interocular separation to face size. This is consistent with the critical role of the eyes in upright face identification [46,47]. Additionally, empirical evidence shows that masking the eyes lowers recognition accuracy more than other features [48]. Another study by Keil [49] conducted an image-based analysis showing that internal facial features, particularly the eyes, contain a concentration of horizontally distributed spatial frequency content, primarily within the low to mid SF range. While this study did not examine human behavioral responses directly, it provides a computational account of why these SF bands may carry critical information for face identification. In the context of our findings, distortions in the mid-SF range likely interfere with the encoding of these horizontally organized facial features, such as interocular distance, contributing to impaired recognition performance. These insights support the interpretation that mid-SF distortions disrupt access to important structural cues used during face recognition.

Research on contrast chimeras has highlighted the importance of the eye region in face recognition [22], demonstrating that certain facial regions play a more significant role in processing, even within the context of holistic face perception. Sormaz’ study [50] further emphasize that, although face recognition is generally holistic, some regions, such as the eyes, may be relatively more crucial. These findings align with the broader framework of holistic face processing and contribute to our understanding of how individualized facial representations are formed.

Our findings can be interpreted in the context of holistic face processing, which involves integrating facial features and their spatial relationships into a unified perceptual representation [23,24]. This process is thought to support the rapid and efficient recognition of upright faces and is often disrupted by manipulations such as inversion or visual distortion. In the present study, distortions affecting mid-spatial frequencies may have impaired recognition by interfering with the structural coherence typically preserved during holistic processing. While we use holistic processing as a framework to interpret these effects, we acknowledge that it is not a unitary mechanism and that spatial frequency manipulations may impact both global and local aspects of face perception. Future work should aim to disentangle these contributions using targeted designs that isolate holistic and part-based processing more directly.

Several studies have explored the relationship between spatial frequency (SF) and face processing, and the key differences between upright and inverted face recognition. Upright faces are thought to recruit more holistic or configural processing, relying predominantly on lower SFs, while inverted faces rely more on high SFs for local feature analysis [45,51,52]. For instance, Goffaux’s study [52] demonstrated that low SF information is crucial for holistic processing, as participants performed significantly worse when low SF information was removed from upright faces, while inverted face recognition was less affected. This foundational work aligns with the general view that upright face recognition is optimized for global processing and is sensitive to the loss of low SF information.

Studies [25,53] suggested similar SF tuning for upright and inverted faces. Gaspar’s study [25] used superimposed filtered noise and found no significant differences in SF tuning between upright and inverted face identity recognition, indicating that both orientations may rely on similar SF bands for performance. Similarly, Willenbockel [53] reported overlapping SF tuning curves for upright and inverted faces, raising questions about the specificity of SF tuning differences for global and local processing. These studies suggest that upright and inverted faces may recruit similar SF information under certain conditions, which contrasts with the widely accepted holistic-local distinction.

In our study, the use of warping-based distortions may account for the observed differences from prior findings. Unlike filtered noise or SF filtering techniques used in the studies, warping manipulations disrupt spatial relationships and introduce orientation and configural distortions across SF bands. These warping effects may disproportionately affect upright face recognition by impairing the integration of global low SF information critical for holistic processing. For inverted faces, which rely more on local feature analysis, the impacts of these distortions may be more uniform across SF bands, consistent with the lack of SF dependence observed in our results.

The methodological differences between our study and previous work likely explain the divergent findings. By directly altering spatial relationships and configural information, our approach taps into a different aspect of face perception, potentially disrupting mechanisms that are more sensitive to global processing. Future studies could compare warping distortions with traditional SF filtering to further explore how these manipulations uniquely affect upright and inverted face recognition. These comparisons would clarify whether our results reflect a distinct processing mechanism elicited by warping or a broader characteristic of spatial frequency interactions in face perception.

A related experiment studied how subjects can recognize familiar faces by applying visual distortion to face images, using a parametric model of prosopometamorphopsia [13]. By varying viewing distance, they examined the effect of distortion frequency in cycles per degree of visual angle and cycles per face. They concluded that cycles per face was the critical parameter, as differences in tuning functions at different distances and sizes collapsed onto a single function when expressed in cycles per face. Their study demonstrated that the effect of distortion is influenced by both the spatial frequency of the distortion and the size of the visual stimulus. Our experiment with upright faces supports their findings, which highlight a significant relationship between spatial frequency and the severity of distortion. Our experiment’s novelty further lies within its use of unfamiliar faces, the inversion task, and our image processing that utilized full color with control of chrominance and luminance.

There are several possible explanations for the FIE. The configural-processing hypothesis (CPH) is a popular explanation of the FIE, suggesting that inversion disrupts the spatial arrangement of facial features, which corresponds to configural information [35]. Our data may suggest that recognizing upright faces relies more on configural information (low SF), as the threshold for matching inverted faces increases with increasing distortion (i,e, participants could tolerate more distortion at higher distortion spatial frequencies that low). The CPH is consistent with the presence of the asymmetric U-shaped tuning curve in the upright faces. The tuning function means that configural processing is scale-dependent, it’s not just the total magnitude of feature shifts, it’s the relative scale. This means that moving features relative to one another at the scale of the eyes severely degrades facial recognition abilities. Analogously, this tuning function is not present in inverted faces, indicating that subjects most likely did not prioritize configural information to recognize inverted faces. The tuning curve for face matching suggests that high spatial frequencies, which carry featural information, play a greater role in recognizing inverted faces.

However, Rossion [23] presents a comprehensive framework for understanding holistic face processing, proposing that the face inversion effect (FIE) reflects a disruption in the global, integrative processing of faces. Unlike local or feature-based approaches, holistic processing involves perceiving the face as a unified whole, integrating spatial relationships and overall configuration. Rossion argues that inversion impairs this process by disrupting the neural mechanisms that rely on upright orientation for efficient face recognition. The study also highlights how inversion disproportionately affects the processing of configural information, leading to increased reliance on featural cues. This framework provides a valuable theoretical context for interpreting how manipulations, such as spatial frequency distortions, may differentially affect upright and inverted faces by modulating holistic processing mechanisms. These results elucidate that both featural and configural processing may be present.

Traditionally, face perception research has often distinguished between featural processing, which involves recognizing individual facial features, and configural processing, which involves perceiving the spatial relationships among those features. While this distinction provides a useful conceptual framework, it is important to recognize that it is a simplification of more complex perceptual mechanisms. In particular, our study uses this framework cautiously to interpret how distortions at different spatial frequency bands might differentially affect face recognition. Low spatial frequency distortions are generally thought to impact configural information by altering the global arrangement of facial features, whereas high spatial frequency distortions tend to affect fine details of individual features. Our stimulus manipulations show that distortions at low spatial frequencies can cause substantial warping of local facial features, indicating that the link between spatial frequency and the type of perceptual disruption is more nuanced than a simple division between featural and configural processing. We therefore interpret our findings within this conceptual context while acknowledging their limitations and the need for more integrative models of face processing.

While high SF distortions primarily affect local features, low SF distortions likely alter both feature spatial relationships and local properties, face recognition is also highly orientation-dependent, with horizontal information playing a critical role in identity recognition [49]. The warping technique employed in our study introduces distortions across multiple spatial scales, which may also disrupt orientation content. This distortion of horizontal structure could disproportionately affect face identity recognition, given its reliance on horizontally tuned mechanisms [49]. Future work could further investigate the interplay between spatial frequency and orientation distortions, for example, by isolating orientation-specific content to examine its role in face recognition under different distortion conditions.

While previous studies have examined the role of spatial frequency in face recognition using filtering or masking techniques [25,45] the present study contributes a novel perspective by using spatial warping to distort faces at different frequency bands. This approach disrupts both structural and orientation content in a way that more closely resembles visual distortions seen in clinical conditions like prosopometamorphopsia. Unlike prior research, our results reveal a strong sensitivity to mid-SF distortions specifically in upright face recognition, a pattern not evident in inverted faces, and show that this effect is robust across individuals. These findings extend the existing literature by highlighting the critical role of distortion type and orientation in shaping how spatial frequency information is used during face recognition.”

Understanding the mechanisms behind facial recognition helps with understanding why humans sometimes fail when processing faces. Many populations are affected by prosopagnosia including otherwise normally-sighted individuals, neurodivergent people, and patients with visual abnormalities. With these atypical populations, standard methods are only able to confirm whether there are selective face-processing deficits, but they do not interrogate why, how severe they are, or suggest approaches that may be effective to rehabilitate them. So, to disentangle and better understand facial recognition performance, our method using distortion provides an assumption-free approach. It allows for systematic control of image manipulations and from the subjects’ results, we can analyze and draw conclusions based on unbiased data. Whereas many face perception studies have employed selective alterations like swapping a nose or modifying interocular distances, thus pre-biasing the data by assuming the alterations are what is driving face recognition. Our method improves upon some previous face perception literature by using inclusive and diverse stimuli and techniques like matching image chrominance and luminance.
